# A Mendelian randomization investigation of the causal association between the gut microbiota and sleep disorders

**DOI:** 10.3389/fmicb.2024.1372827

**Published:** 2024-03-22

**Authors:** Wei Yan, Zhenzhen Zhuang, Yuhao Gao, Yuntao Wang, Daikun He

**Affiliations:** ^1^Department of General Practice, Jinshan Hospital, Fudan University, Shanghai, China; ^2^Department of General Practice, Zhongshan Hospital, Fudan University, Shanghai, China; ^3^Center of Emergency and Critical Care Medicine, Jinshan Hospital, Fudan University, Shanghai, China

**Keywords:** causal association, gut microbiota, gut-brain axis, Mendelian randomization, sleep disorders

## Abstract

**Background:**

Increasing numbers of people are suffering from sleep disorders. The gut microbiota of these individuals differs significantly. However, no reports are available on the causal associations between specific gut microbiota and sleep disorders.

**Methods:**

Data on gut genera were obtained from the MiBioGen consortium. Twenty-four cohorts with 18,340 individuals of European origin were included. Sleep disorder data, which included 216,454 European individuals, were retrieved from the FinnGen Biobank. Subsequently, two-sample Mendelian randomization was performed to analyze associations between sleep disorders and specific components of the gut microbiota.

**Results:**

Inverse variance weighting (IVW) revealed a negative correlation between *Coprobacter* and sleep disorders (OR = 0.797, 95% CI = 0.66–0.96, and *p* = 0.016), a positive correlation between *Lachnospiraceae* and sleep disorders (OR = 1.429, 95% CI = 1.03–1.98, and *p* = 0.032), a negative association between *Oscillospira* and sleep disorders (OR = 0.745, 95% CI = 0.56–0.98, and *p* = 0.038), and a negative association between *Peptococcus* and sleep disorders (OR = 0.858, 95% CI = 0.74–0.99, *p* = 0.039).

**Conclusion:**

A significant causal relationship was found between four specific gut microbiota and sleep disorders. One family, *Lachnospiraceae*, was observed to increase the risk of sleep disorders, while three genera, namely, *Coprobacter, Oscillospira*, and *Peptococcus*, could reduce the risk of sleep disorders. However, further investigations are needed to confirm the specific mechanisms by which the gut microbiota affects sleep.

## Introduction

Adequate sleep is critical to both physical and mental wellbeing (Wang et al., 2015). Many studies have shown that an increasing number of people are experiencing varying degrees of sleep disorders, with the prevalence of such disorders increasing with age and exhibiting a trend toward affecting younger individuals (Ford et al., 2015). Sleep disorders affect physical and mental health and lead to cardiovascular diseases, endocrine disorders, and mental illnesses (Reutrakul and Van Cauter, 2018; Huang et al., 2020; Malhotra, 2022), which significantly affect people’s physical health and daily life. Therefore, exploring the mechanisms and treatment of sleep disorders is crucial. Current studies have revealed a complex association between sleep disorders and intestinal microorganisms (Matenchuk et al., 2020). Furthermore, differences in microbiota compositions have been observed in people with short sleep durations relative to those with normal sleep durations (Agrawal et al., 2021). Additionally, animal experiments have shown that prolonged disturbances in the gut microbiota in mice resulting from antibiotic use lead to abnormal sleep patterns (Ogawa et al., 2020), indicating an association between the two. However, no specific causal relationship has been identified. Thus, in this study, Mendelian randomization (MR) was used to assess a possible causal association between disordered sleep and the composition of the gut microbiota, with the findings providing evidence for such a relationship.

As an inferential method for investigating causal relationships, MR relies on Mendel’s principles of heredity and single-nucleotide polymorphisms (SNPs) or genetic variations as instrumental variables for inferring causal associations between exposure factors and outcomes (Davey Smith and Hemani, 2014). The use of MR can reveal underlying biological mechanisms, avoid interference from confounding factors, and thus ensure the accuracy of causal relationships (Birney, 2022).

## Methods

### Data source

Genetic data on intestinal microorganisms were retrieved from the latest pooled data in the MiBioGen consortium.^1^ The present investigation was a genome-wide association study (GWAS) that included 24 cohorts with a total of 18,340 individuals. The gut microbiota data included 211 microbial taxa, classified at six levels, from phylum to species. Microorganisms were identified by analysis of V4, V3–V4, and V1–V2 within the 16S rRNA gene, together with mapping of quantitative trait loci (Kurilshikov et al., 2021).

The genetic data for sleep disorders were retrieved from the latest pooled data of the GWAS from the FinnGen Biobank,^2^ which began in 1975 and included 216,454 individual European participants through 2015. The eligibility criteria were nonorganic sleep disorders diagnosed according to ICD-10 code F51 including insomnia, hypersomnia, sleep–wake rhythm disorders, night terror, and nightmares. Exclusion criteria: (1) any mental disorder, or mental disorder or neurological disease complicated by pregnancy, delivery, or puerperium; (2) sleep disorders combined any disease. After screening, 2,628 participants were selected for the analysis, all of them were European, including 1,415 male and 1,213 female, with an average age of 44.34 years, and the prevalence of sleep disorders was 1.21%. The key figures are shown in Table 1.

**Table 1:** The key figures of the study.

	All	Female	Male
Number of individuals	2,628	1,213	1,415
Unadjusted period prevalence (%)	1.21	1.15	1.29
Median age at first event (years)	44.34	43.10	45.80
Ethnicity	European	European	European

### Instrumental variable (IV) selection

The IVs were selected according to four criteria. First, the significance threshold for the gut microbiota was set at *p* < 5.0 × 10^−8^, Unfortunately, only a limited number of gut microbiota were chosen as instrumental variables after SNP selection. To obtain more comprehensive results and explore further relationships between sleep disorders and gut microbiota, we employed threshold (p < 5.0 × 10^−5^) to identify SNPs below the level of whole-locus significance in order to discover additional potential causal associations. Second, the minimum allele frequency threshold for meaningful variation was 0.01. The principle of the MR Method is that there is no linkage disequilibrium in the IVs analyzed, as the presence of a strong linkage disequilibrium may bias the results. In this study, the independence of the gut microbiota was set at *R*^2^ < 0.001, KB = 10,000. Third, in MR analysis, the most important step is to ensure that the SNP responds to the same alleles as the influence of the exposure factor and the outcome factor. To avoid chain orientation distortion or allelic coding, we removed palindromic SNPS from the data. Fourth, potential pleiotropic effects were examined using MR-PRESSO and MR–Egger regression, resulting in the exclusion of outliers (Bowden et al., 2019). Moreover, MR analyses were conducted under three assumptions, namely, that there was an association between the IVs and exposure factors but not between the IVs and confounding factors and that the effects of the IVs on outcomes were mediated only by the exposure factors. Furthermore, the F-statistic for SNPs was determined for the evaluation of weak instrument bias (Burgess et al., 2011). Specifically, an F-statistic value ≥10 is indicative of an absence of weak instrumental variables, while a value of <10 suggests that weak instrumental variables should be excluded. The F-statistic for relevant SNPs can be found in Additional File 1.

### Study design

**Figure 1:**
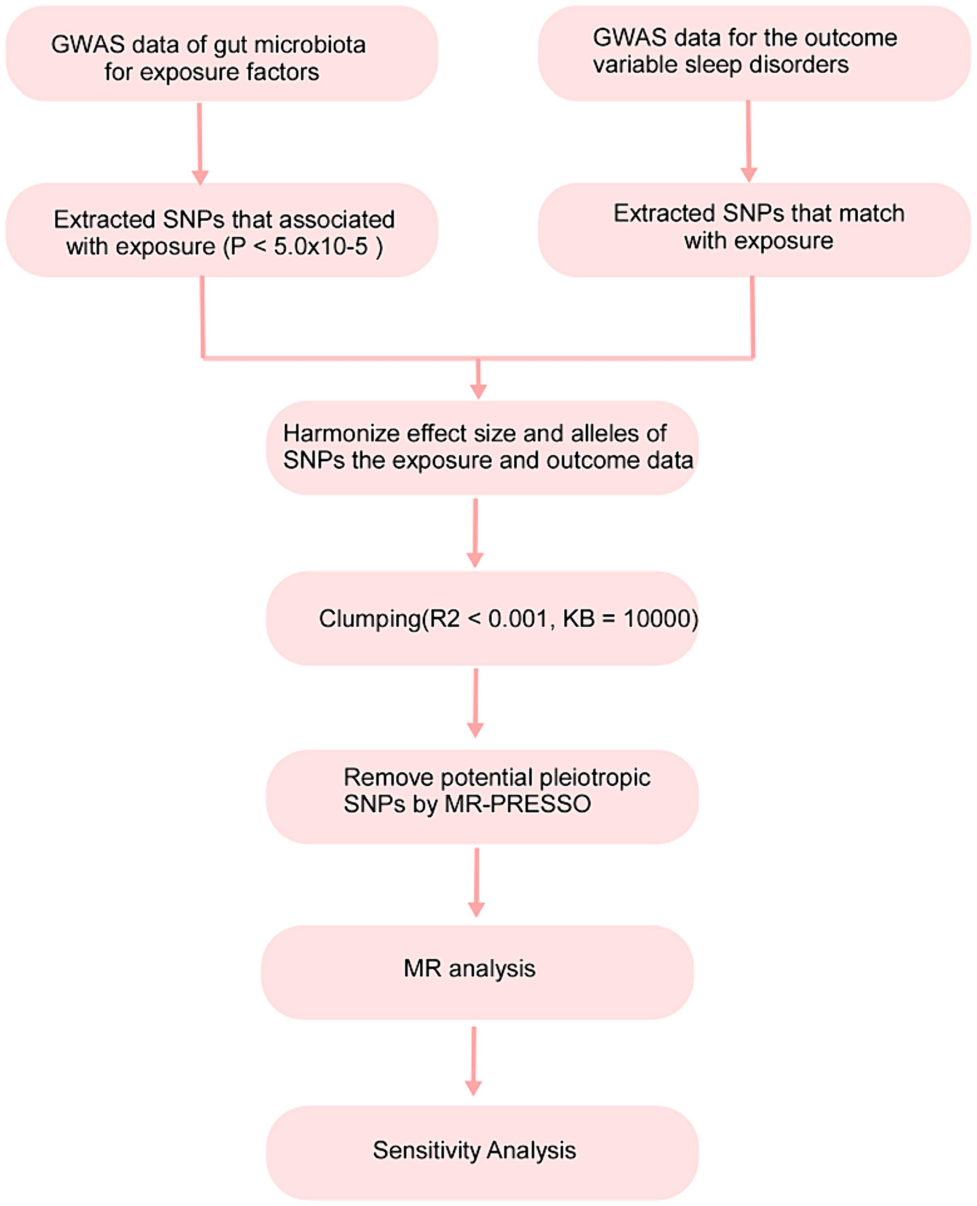
MR analysis flow chart.

In this study, 211 gut microbiota constituents were selected as exposure factors, and sleep disorders were considered the outcome. The flowchart of the study process is shown in Figure 1.

### Statistical analysis

Statistical analyses were performed with MR-PRESSO 1.0, RStudio 4.2.1, and TwoSampleMR 0.5.6, and the IVW method was mostly used for statistical testing due to its greater efficiency in detection. Additional methods, including the weighted median estimation (WME), MR–Egger regression, MR-PRESSO, simple mode (SM), and weighted mode (WM) methods, were also used. These tests were chosen for several reasons. First, IVW is commonly used for MR with multiple IVs, assumes that genetic variants represent relevant IVs, and is capable of detecting causal associations (Bowden et al., 2017). Second, MR–Egger regression was used to analyze summary data, assuming that genetic variation has horizontal pleiotropy. This method estimates the intercept of IVs through weighted linear regression with the presence of an intercept (Burgess and Thompson, 2017). Third, MR-PRESSO estimates the degree of horizontal pleiotropy by adding residuals for each SNP, correcting for horizontal pleiotropy, and obtaining IVW results after correction. Fourth, the WME corrects for the effect of invalid IVs with robust estimates even in situations with 50% invalid IVs. Finally, a leave-one-out strategy was used, in which one SNP was removed at a time before the influence of the remaining SNPs on the outcome was calculated, thereby assessing the effects of outliers. The direction of causal relationships was further identified through reverse MR analysis.

## Results

### Results of MR

**Figure 2:**
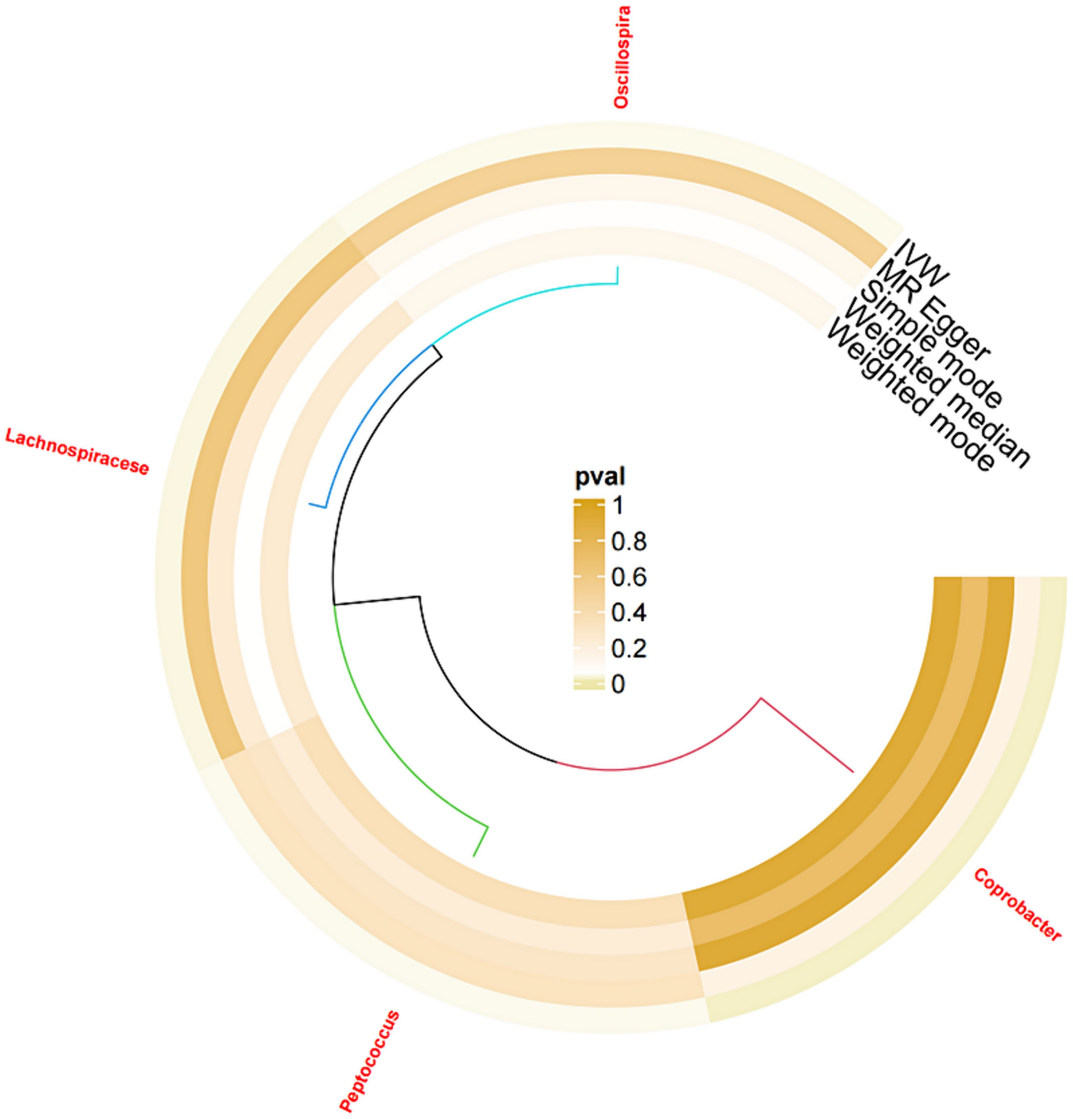
Ring diagram of the gut microbiota associated with sleep disorders.

After significance and correlation analyses were performed for 211 types of gut microbiota, 2,561 relevant SNPs were selected as IVs. Subsequently, a two-sample MR analysis was conducted on sleep disorder outcome data, which revealed 51 significantly associated SNPs, as detailed in Table 2. Analysis of positive SNPs using PhenoScanner did not reveal any associations with confounding factors. Ultimately, four types of gut microbiota were found according to IVW (*p* < 0.05). As shown in Figure 2.

**Table 2:** MR analysis of the samples.

Exposure	Method	No. of SNP	*P*	OR	95% CI	*F*-statistic
Coprobacter	IVW	13	0.017	0.797	0.66–0.96	912.39
	MR–Egger		0.175	1.543	0.86–2.77	
	SM		0.929	0.979	0.63–1.53	
	WME		0.720	0.953	0.73–1.24	
	WM		0.924	0.979	0.64–1.49	
Lachnospiraceae	IVW	13	0.032	1.429	1.03–1.98	363.24
	MR–Egger		0.622	1.437	0.35–5.83	
	SM		0.238	1.493	0.79–2.81	
	WME		0.052	1.502	0.99–2.26	
	WM		0.253	1.482	0.78–2.82	
Oscillospira	IVW	9	0.038	0.745	0.56–0.98	364.31
	MR–Egger		0.514	1.529	0.45–5.13	
	SM		0.135	0.623	0.36–1.08	
	WME		0.065	0.719	0.51–1.02	
	WM		0.133	0.632	0.37–1.08	
Peptococcus	IVW	16	0.039	0.858	0.74–0.99	1229.53
	MR–Egger		0.328	0.758	0.44–1.29	
	SM		0.298	0.836	0.60–1.16	
	WME		0.227	0.883	0.72–1.08	
	WM		0.354	0.856	0.62–1.18	

MR, Mendelian randomization; No. of SNP is the number of SNPs being used as IVs; IVW, inverse variance weighted; SM, simple mode; WME, weighted median estimator; WM, weighted mode; OR, odds ratio; CI, confidence interval.

### Detailed MR analysis results

**Figure 3:**
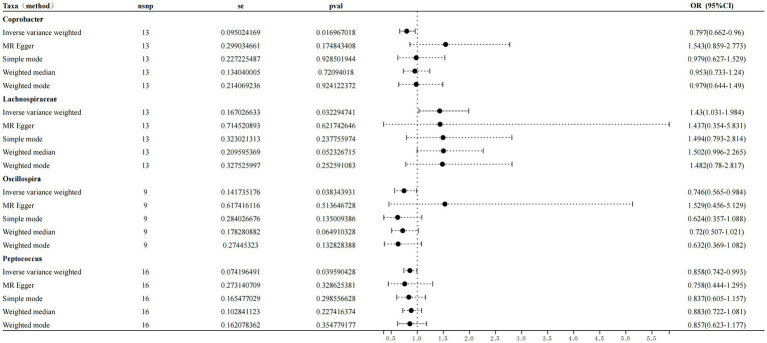
Forest plot showing the influence of intestinal microorganisms on sleep disorders; OR, odds ratio; CI, confidence interval.

Following the MR analyses, IVW indicated that *Lachnospiraceae* (OR = 1.429, 95% CI = 1.03–1.98, *p* = 0.032) increased the risk of sleep disorders, whereas *Coprobacter* (OR = 0.797, 95% CI = 0.66–0.96, and *p* = 0.016), *Oscillospira* (OR = 0.745, 95% CI = 0.56–0.98, and *p* = 0.038), and *Peptococcus* (OR = 0.858, 95% CI = 0.74–0.99, and *p* = 0.039) reduced the risk of sleep disorders. The details are provided in the forest plot (Figure 3).

### Sensitivity analyses

**Figure 4:**
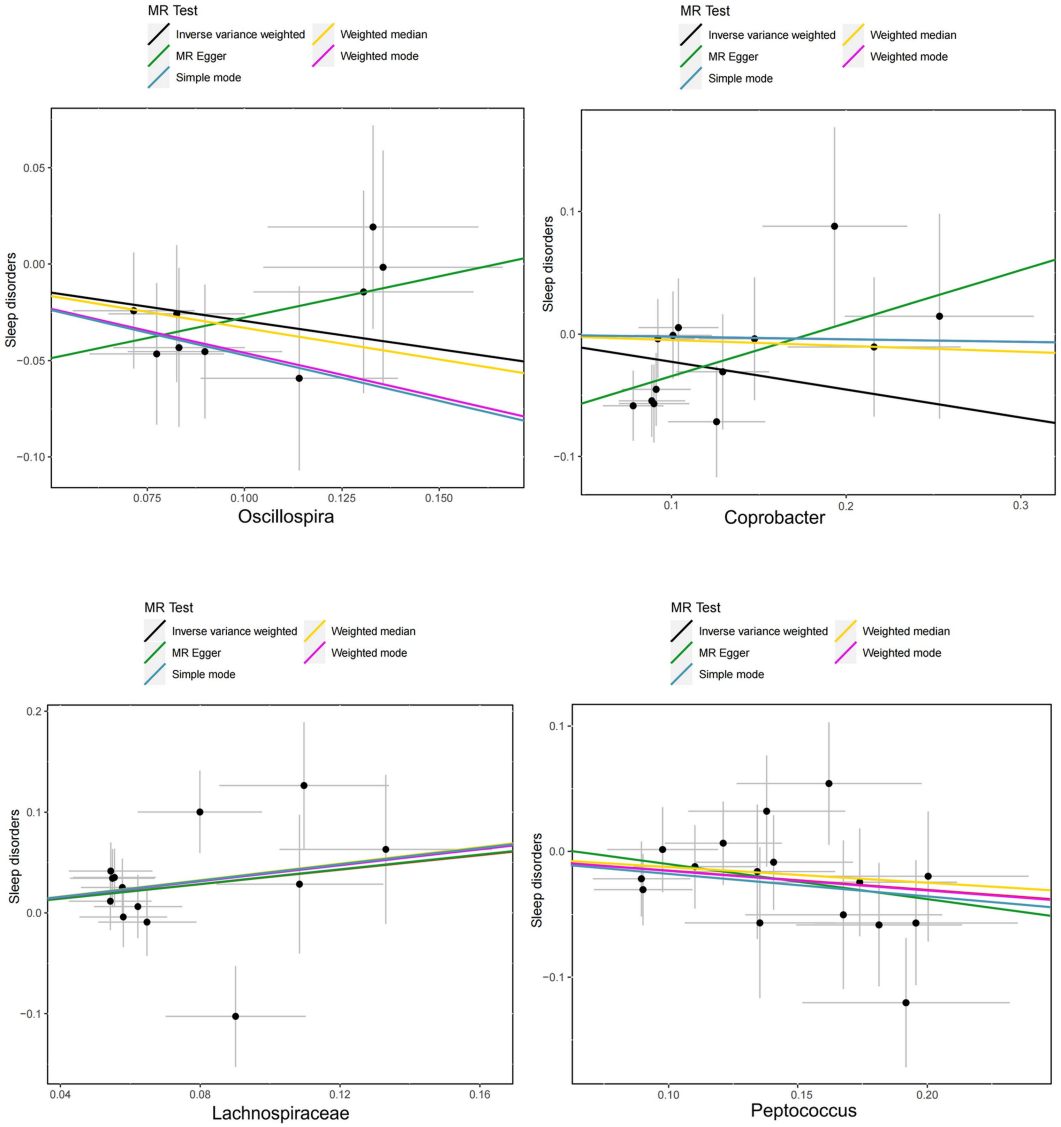
Scatter plots of the influence of the intestinal microbiota on sleep disorders.

**Figure 5:**
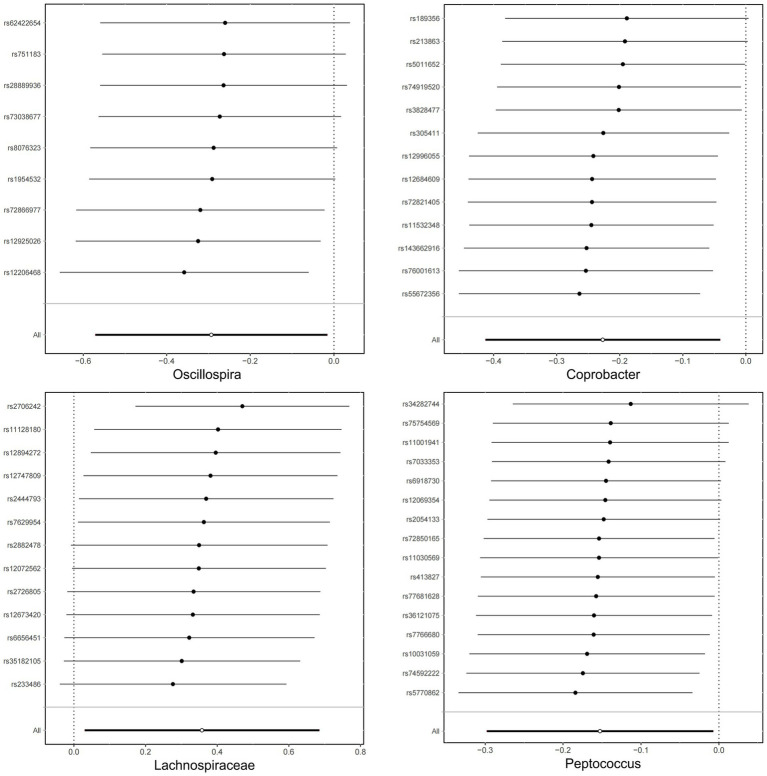
Leave-one-out plots for analyzing the influence of the intestinal microbiota on sleep disorders.

The scatter plot (Figure 4) shows consistent directions for the remaining two gut microbiota, except for *Oscillospira* and *Coprobacter*. Owing to the strong statistical power of the IVW method, this was responsible for most of the present findings. No horizontal pleiotropy was found by MR–Egger or MR-PRESSO (sensitivity analysis, *p* > 0.05) using MR–Egger intercept tests or the MR-PRESSO global method (Supplementary Appendix 2). After heterogeneity testing, no significant heterogeneity was identified by the Cochrane Q test (*p* > 0.05). Moreover, the leave-one-out plots (Figure 5) demonstrated that the gradual removal of an SNP had an insignificant effect on the results, indicating a relatively stable causal relationship.

## Discussion

This is an MR study on the causal associations between the intestinal microbiota and sleep disorders. These findings confirmed this relationship, indicating that *Lachnospiraceae* is a risk factor for sleep disorders, as these bacteria markedly increase the risk of such disorders. In contrast, *Coprobacter*, *Oscillospira*, and *Peptococcus* act as protective factors that can significantly reduce the risk of sleep disorders.

Close associations between *Lachnospiraceae* and sleep have been reported. An observational study revealed marked increases in the levels of *Lachnospiraceae* in the intestines of patients with sleep apnea (Zhang et al., 2022). Moreover, a collaborative study by the University of Haifa and Israel Institute of Technology revealed a significant link between the gut microbiota and sleep patterns, indicating that people who stay up late exhibit a significantly greater level of *Lachnospira* in their gut, which is negatively correlated with quality of sleep (Carasso et al., 2021). The findings of the present study are consistent with these results. Currently, the specific mechanism through which *Lachnospiraceae* affects sleep quality has not been determined. Hypothetically, this difference may be related to the regulation of sleep- and wake-related signals by short-chain fatty acids produced by *Lachnospiraceae*.

Interestingly, it was found here that *Coprobacter* can lower the risk of sleep disorders. A clinical study reported a positive association between regional homogeniety (ReHo) values in the left angular gyrus and the Self-rating depression scale (SDS) score, suggesting the potential involvement of this region in the modulation of emotion in individuals with chronic insomnia (Gong et al., 2020). It has also been reported that the ReHo values in this region are positively correlated with the relative abundance (RA) of *Coprobacter* (Feng et al., 2021), suggesting an association between the RA of *Coprobacter* and improved cognition in chronic insomnia (Feng et al., 2021). These results indicate the involvement of intestinal *Coprobacter* abundance in sleep regulation, consistent with the findings of the present investigation. However, the specific mechanism through which *Coprobacter* species affect sleep quality has not been determined. Presumably, this difference may be due to the short-chain fatty acid production by *Coprobacter*, which can affect sleep by improving memory and mood in humans (Smith et al., 2015).

*Oscillospira* is a mysterious gut microbiota widely present in the human intestine that has been linked to a variety of disorders, such as obesity, gallstones, leanness, and chronic constipation (Yang et al., 2021), and represents a potential next-generation probiotic. Moreover, it has been shown that the RA of *Oscillospira* is associated with central nervous system disorders. Specifically, one study revealed a greater incidence of *Oscillospira* in the intestines of Parkinson’s disease (PD) patients than in the intestines of HCs (Zhang et al., 2020), the findings of which contrast with those of another study. In contrast, another clinical study showed reduced intestinal RA of *Oscillospira* in patients with PD (Vascellari et al., 2020); however, a causal relationship has not been confirmed owing to the lack of evidence. Animal studies have shown negative associations between the RA of *Oscillospira* and depression (Zhang et al., 2021). Our study is the first to reveal a negative correlation between *Oscillospira* and sleep disorders, indicating that *Oscillospira* acts as a protective factor against sleep disorders. Although further investigations are needed to determine the underlying mechanism by which *Oscillospira* influences sleep, the present findings suggest a new target for microbial therapy for sleep disorders.

Interestingly, the present study revealed a protective effect of *Peptococcus* on sleep disorders, marking the first report of this relationship. *Peptococcus* is a common anaerobic bacterium in the gut microbiota and is a conditional pathogen that can be isolated from individuals with various suppurative infectious diseases (Bourgault et al., 1980). There are no relevant reports available on the relationship between *Peptococcus* and sleep; hence, additional investigations into the mechanism by which *Peptococcus* affects sleep are needed.

**Figure 6:**
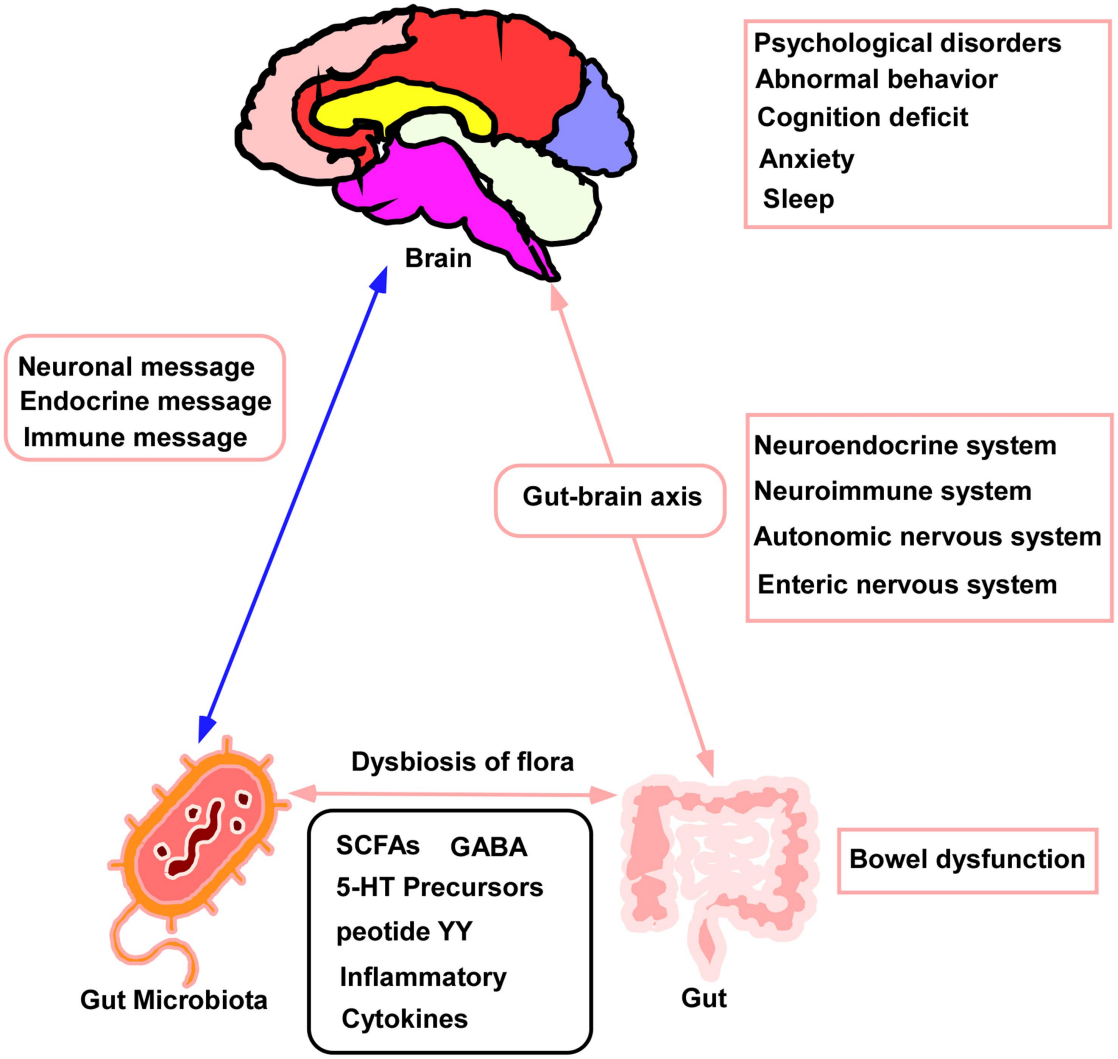
Schematic of the gut–brain axis.

The gut microbiota is a complex ecosystem that has been linked to a variety of systemic diseases. In addition, different gut microbiota produce various metabolites, mainly short-chain fatty acids, inflammatory factors, and other molecules (Schoeler and Caesar, 2019). These metabolites influence metabolic processes in different organs of the body, contributing significantly to their normal function (Nishida et al., 2018). Such interactions are termed “gut-organ axes” and include axes between the gut and kidney, liver, bone, and brain (Ahlawat and Asha, 2021). The gut-brain axis has been described both in animal models and in humans (Góralczyk-Bińkowska et al., 2022) and involves the central nervous system, the autonomic and enteric nervous systems and the hypothalamic–pituitary–adrenal axis (Mayer et al., 2022). It is influenced by metabolites produced by intestinal microorganisms that transmit signals between the gastrointestinal tract and the nervous system, modulating both behavior and disease (Mörkl et al., 2020). Figure 6 shows a schematic of the gut-brain axis. The gut microbiota can thus modulate the nervous system and behavior through this axis, thereby affecting sleep.

## Conclusion

MR analysis indicated that *Lachnospiraceae* may increase the risk of sleep disorders, while sleep disorder risk may be reduced by *Coprobacter, Oscillospira*, and *Peptococcus*. However, the underlying mechanisms by which these intestinal microorganisms affect sleep quality remain unclear, and further investigation is needed. These findings suggest that the gut microbiota may represent a target for treating sleep disorders.

The advantage of this study is that the causal relationship between exposure factors and outcomes is analyzed at the genetic level, so that the association obtained is not affected by causal inversion and is not affected by confounding factors. It can overcome some limitations and problems faced by traditional observational studies and randomized controlled trial (RCT).

This study also has some limitations. Firstly, the GWAS data obtained are from European populations, which is not universal enough for people with different ethnic genetic backgrounds. Secondly, the 16S test used in the intestinal flora data in this study may not be complete due to technical reasons, and the number of intestinal flora tested may not be complete, and whole genome testing may be required; Finally, there may be false positive or false negative results due to the screening and verification of genetic variation. The accuracy of analysis results is affected.

## Data availability statement

The datasets presented in this study can be found in online repositories. The names of the repository/repositories and accession number(s) can be found in the article/Supplementary material.

## Ethics statement

The GWAS data used in this investigation were obtained from a public database, and the preliminary experiments were conducted with informed consent and ethical approval from relevant institutions. Thus, the need for further ethical approval was waived.

## Author contributions

WY: Conceptualization, Data curation, Formal analysis, Funding acquisition, Methodology, Software, Writing – original draft, Writing – review & editing, Investigation. ZZ: Data curation, Methodology, Writing – original draft. YG: Data curation, Software, Writing – original draft. YW: Formal analysis, Software, Writing – original draft. DH: Writing – review & editing.

## Figures and Tables

**Table:** 

IVW	Inverse variance weighting
MR	Mendelian randomization
SNPs	Single-nucleotide polymorphisms
GWAS	Genome-wide association study
IV	Instrumental variable
WME	Weighted median estimation
SM	Simple mode
WM	Weighted mode
OR	Odds ratio
CI	Confidence interval
RA	Relative abundance
PD	Parkinson’s disease
ReHo	Regional homogeniety
SDS	Self-rating depression scale
RCT	Randomizedcontrolledtrial

## Footnotes

^1^ https://mibiogen.gcc.rug.nl

^2^ https://r5.finngen.fi//
